# PR Domain-containing Protein 7 (PRDM7) Is a Histone 3 Lysine 4 Trimethyltransferase*

**DOI:** 10.1074/jbc.M116.721472

**Published:** 2016-04-29

**Authors:** Levi L. Blazer, Evelyne Lima-Fernandes, Elisa Gibson, Mohammad S. Eram, Peter Loppnau, Cheryl H. Arrowsmith, Matthieu Schapira, Masoud Vedadi

**Affiliations:** From the ‡ Structural Genomics Consortium, University of Toronto, Toronto, Ontario M5G 1L7,; the § Princess Margaret Cancer Centre and Department of Medical Biophysics, University of Toronto, Toronto, Ontario M5G 2M9, and; the ¶ Department of Pharmacology and Toxicology, University of Toronto, Toronto, Ontario M5S 1A8, Canada

**Keywords:** epigenetics, histone methylation, histone modification, substrate specificity, zinc finger

## Abstract

PR domain-containing protein 7 (PRDM7) is a primate-specific histone methyltransferase that is the result of a recent gene duplication of PRDM9. The two proteins are highly homologous, especially in the catalytic PR/SET domain, where they differ by only three amino acid residues. Here we report that PRDM7 is an efficient methyltransferase that selectively catalyzes the trimethylation of H3 lysine 4 (H3K4) both *in vitro* and in cells. Through selective mutagenesis we have dissected the functional roles of each of the three divergent residues between the PR domains of PRDM7 and PRDM9. These studies indicate that after a single serine to tyrosine mutation at residue 357 (S357Y), PRDM7 regains the substrate specificities and catalytic activities similar to its evolutionary predecessor, including the ability to efficiently methylate H3K36.

## Introduction

The PRDM^3^ (PRDI-BF1 and RIZ homology domain) family of proteins is defined based on the conserved N-terminal PR domain. PR domains are closely related to the Su(var)3–9, enhancer of zeste, and trithorax (SET) domains of histone methyltransferases (1–3). The PRDM family consists of 17 members in primates. Some PRDMs such as PRDM1, -2, -5, and -12 act as tumor suppressors and some others such as PRDM3, -13, -14, and -16 are oncogenic (reviewed in Ref. 4). In addition, PRDM1, -3, -5, and -16 have been implicated in a variety of other diseases, including systemic lupus erythematosus (5), rheumatoid arthritis (6), lung dysfunction (7), human sterility (8), and migraine (9). PRDMs play diverse roles in cell-cycle regulation, differentiation, and meiotic recombination (4).

Although methyltransferase activity has been reported for PRDM2, -3, -6, -8, -9, and -16 (10–16), the functionality of the PR domain of other PRDM family members remains to be discovered. Included in this HMT-active subset of PRDM proteins is PRDM9, a H3K4 methyltransferase (15, 17) that recently has been reported to efficiently mono-, di-, and trimethylate H3K36 as well as H3K4 *in vitro* and in cells (14). Physiologically, PRDM9 is intimately involved in meiotic recombination (18–21) and is an important speciation factor in mammals (22–26). It is selectively expressed in cells undergoing meiosis, and genetic deletion of the *PRDM9* gene results in defective gametogenesis and sterility (15). To our knowledge, PRDM9 is currently the only PRDM family member for which detailed enzyme kinetics have been reported (14). It is possible that other PRDMs require one or more interacting partners for histone methyltransferase activity, or perhaps they methylate non-histone targets. Most PRDM family proteins contain a variable number of C2H2 zinc finger repeats that may contribute to their interaction with DNA or protein partners in the cell. Some PRDMs act as scaffolding proteins by binding to DNA via these zinc finger motifs to recruit transcription factors to target gene promoters (reviewed in Refs. 27 and 28). In some cases these interactions may be essential for methyltransferase activity. Interestingly, certain PRDM isoforms lack the PR domain (29, 30), suggesting that some PRDM proteins may function predominantly as scaffolding proteins. It also raises the intriguing possibility that PR domains that lack HMT activity may instead function as “reader” domains to further facilitate proper genomic localization.

**FIGURE 1.**
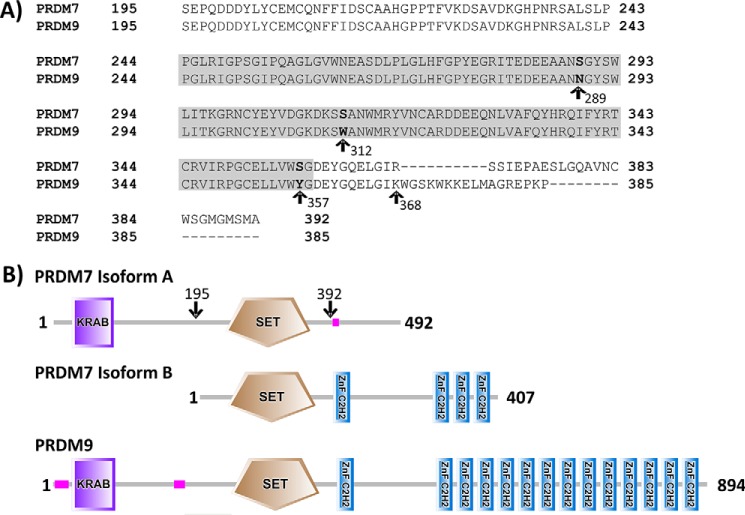
**Alignment of the PRDM7(195–392) construct studied in this article and the PRDM9(195–385) construct described by Eram *et al.* (14).** *A,* the PR domains of human PRDM7 (Q9NQW5) and PRDM9 (Q9NQV7) were defined per the Uniprot annotation (*gray box*, residues 244–358 for both proteins). The three divergent residues in the PR domain between the two sequences are indicated. The start of the C-terminal divergence between the constructs is noted by the *arrow* at position 368. *B,* domain architecture of the canonical isoform of PRDM7 (isoform A) and a secondary isoform of PRDM7 (isoform B) that contain 0 or 4 zinc fingers, respectively. PRDM9 is also shown for reference. *Arrows* indicate the approximate construct boundaries of the PRDM7 (isoform A) recombinant protein used for enzymatic assays. Proteins are represented by SMART protein annotation.

In primates, a recent gene duplication of *PRDM9* has resulted in the creation of *PRDM7* (30). PRDM7 is highly homologous with its ancestral gene product, sharing an amino acid sequence identity of 41% overall and 97% over the PR domain (Fig. 1*a*). Indeed, there are only three divergent residues between the PR domains of PRDM7 and PRDM9 (residues 244–358 in both proteins). Outside of the PR domain, PRDM7 has experienced major structural rearrangements with 0 or 4 zinc finger repeats (depending upon the isoform) *versus* 14 in PRDM9, and modified gene splicing due to tandem duplication of an 89-nucleotide long segment from ancestral exon 3 that codes for the C-terminal part of PR domain (Fig. 1*b*) (30). PRDM9 is the only member of the PR domain family whose expression is restricted to germ cells entering meiotic prophase (15). Although PRDM7 maintains some tissue specificity, it is more broadly expressed than PRDM9 (30).

In this study we have fully characterized the enzymatic properties of PRDM7. We show that PRDM7 is an active methyltransferase with different substrate specificity than that of the highly homologous PRDM9.

## Experimental Procedures

### 

#### 

##### Chemicals

[^3^H]*S*-Adenosylmethionine ([^3^H]AdoMet), containing the ^3^H radiolabel on the labile methyl group, was obtained from PerkinElmer Life Sciences (catalog number NET155V001MC, Specific activity range 12–18 Ci/mmol). *S*-adenosylmethionine (AdoMet) was obtained from AK Scientific (Union City, CA). Biotinylated peptide substrates were obtained from Peptide2.0 (Chantilly, VA) or Tufts University Peptide Synthesis Core Facility (Boston, MA). All other chemicals were obtained from Sigma or LifeTech and were reagent grade or higher. Peptide sequences were as follows: H3(1–25), ARTKQTARKSTGGKAPRKQLATKAA-GK-biotin; H3K4A, ARTAQTARKSTGGKAPRKQLATKAA-GK-biotin; H3K4me1, ART(Kme1)QTARKSTGGKAPRKQLATKAA-GK-biotin; H3K4me2, ART(Kme2)QTARKSTGGKAPRKQLATKAA-GK-biotin; H3(21–44), ATKAARKSAPATGGVKKPHRYRPG-GK-biotin; H3K36me1, ATKAARKSAPATGGV(Kme1)KPHRYRPG-GK-biotin; H3K36me2, ATKAARKSAPATGGV(Kme2)KPHRYRPG-GK-biotin.

##### Protein Expression and Purification

A synthetic construct encoding for amino acids 1–482 of human PRDM7 was prepared by GenScript. A DNA fragment corresponding to the SET domain of PRDM7 (amino acid residues 195–392) was cloned into the pET28-MHL vector (Addgene plasmid number 26096) using the In-Fusion® cloning kit (Clontech). These boundaries were selected to optimize protein expression and solubility. Single-point PRDM7 mutants S289N, S312W, S357Y, and the triple mutant (S289N/S312W/S357Y) were generated by site-directed mutagenesis. Site-directed mutagenesis on a previously described PRDM9(195–385) construct (14) was performed to generate PRDM9 Y357S. A construct containing residues 195–367 of PRDM7 appended with residues 368–385 of PRDM9 was generated by amplifying the desired region of PRDM7 from the pET28-MHL construct using extended primers to introduce the PRDM9 sequence. A construct containing residues 195–367 of PRDM9 appended with residues 368–392 of PRDM7 was generated using a similar approach by using PRDM9(195–385) pET28-MHL as a template. Recombinant protein was overexpressed as a hexahistidine tag fusion in *Escherichia coli* strain BL21(DE3) V2R-pRARE2 during an overnight induction with 0.5 mm isopropyl 1-thio-d-galactopyranoside at 18 °C. Cells were resuspended in 20 mm Tris-HCl (pH 7.5), 500 mm NaCl, 5% glycerol, 5 mm imidazole. Chemical lysis was performed by rotating the cells for 30 min at 4 °C after the addition of 0.5% CHAPS, benzonase nuclease, 1 mm PMSF, 1× cOmplete EDTA-free protease inhibitor mixture tablet (Roche Applied Science, Penzberg, Germany), and 2 mm β-mercaptoethanol followed by sonication for 5 min using a 50% duty cycle (10 s on/10 s off) at a power setting of 8 (Sonicator 3000, Misoni). The resulting lysate was clarified by centrifugation for 1 h at 38,400 × *g* at 4 °C. Clarified lysate was loaded onto a Hispur^TM^ nickel-nitrilotriacetic acid column (Thermo Scientific) and washed with 20 mm Tris-HCl (pH 7.5), 500 mm NaCl, 5% glycerol, 5 mm imidazole followed by a second wash with the same buffer containing 15 mm imidazole. Retained protein was eluted with the same buffer containing 250 mm imidazole and 0.5 mm tris(2-carboxyethyl)phosphine hydrochloride (TCEP). The recovered protein was then concentrated, and further purified over a Superdex 200 26/60 size exclusion column in a running buffer consisting of 20 mm Tris-HCl (pH 8.0), 300 mm NaCl, 5% glycerol, and 0.5 mm tris(2-carboxyethyl)phosphine. Recovered protein was concentrated and purity was determined by SDS-PAGE and LC-MS.

##### Differential Scanning Fluorimetry

Experiments were performed as previously described (31). Briefly, proteins were diluted to 0.24 g/liter in 20 mm Bis-tris propane (pH 8.0) in the presence of 5× SYPRO Orange (Life Technologies) dye in a 384-well white PCR plate (Axygen, number PCR-384-W). To this mixture was added AdoMet or a pH-matched vehicle control and fluorescence (excitation 465/emmission 580) was continuously monitored over a 25–95 °C temperature gradient at a rate of 4 °C/min using a Light Cycler 480 II Instrument (Roche). *T_m_* values were calculated from Boltzmann regression analysis using Bioactive (version 2.1.10) software and analyzed using GraphPad Prism (version 6.03).

##### Mass Spectral and Western Blotting Analysis of Histone Peptide and Nucleosome Methylation

End point methylation reactions were performed in a buffer containing 60 mm Bis-tris propane (pH 9.0) supplemented with 10 mm DTT in a total volume of 40 μl. Histone peptides (250 μm) were incubated overnight (∼16–18 h) in the presence of 3 mm AdoMet with or without PRDM proteins (1 μm for WT, S289N, S312W; 0.1 μm for S357Y) at 22 °C. Reactions were quenched by the addition of 110 μl of 0.1% trifluoroacetic acid and analyzed using an Agilent LC/MSD Time-of-Flight Mass Spectrometer equipped with an electrospray ionization source. To remove buffer components from the reaction mixture, the samples were separated over a POROSHELL 300SB-C3 HPLC column over a 5–95% acetonitrile/water gradient. Similarly prepared samples were also subjected to Western blotting analysis as described below, except that all enzymes were used at a concentration of 1 μm. End point methylation reactions with nucleosome as substrate were performed using the same assay conditions as for peptides but at 5 μm PRDM7, 5 mm AdoMet, and 1 μm recombinant nucleosome.

Western blotting was performed using specific antibodies for H3K36me1 (Abcam, ab9048), H3K36me2 (Cell Signaling Technology, 2901), H3K36me3 (Cell Signaling Technology, 9763), H3K4me1 (Abcam, ab8895), H3K4me2 (Millipore, 07030), and H3K4me3 (Abcam, ab8585). The specificity of all H3K4 and H3K36 antibodies were assessed by immunoblotting methylated peptides as previously reported (14, 32).

##### Radiometric Methyltransferase Assays

Radiometric methyltransferase assays were performed using biotinylated peptide substrates and [^3^H]AdoMet in a reaction buffer containing 20 mm Bis-tris propane (pH 9.0) supplemented with 5 mm DTT and 0.01% Triton X-100. Ten microliter reactions were initiated with [^3^H]AdoMet and allowed to proceed at 23 °C before being quenched with excess guanidine hydrochloride. The range of specific activities of AdoMet used in assays is 1.8–0.06 Ci/mmol. For peptide screening, incorporated radioactivity was measured in streptavidin-coated 96-well scintillation proximity FlashPlates Plus (PerkinElmer Life Sciences) in a TopCount NXT plate reader (PerkinElmer Life Sciences). Kinetic constants were measured under pseudo first-order reaction conditions for each protein and incorporated radioactivity was captured on SAM2® Biotin Capture Membranes (Promega, Madison WI) and quantified using liquid scintillation counting. PRDM7 wild-type kinetic parameters for AdoMet and H3K4me2 were determined using a bi-substrate matrix assay format and analyzed using the Enzyme Kinetics module in SigmaPlot version 11.0. Due to the extremely high *K*_*m*_^app^ of AdoMet for wild-type PRDM7, other apparent peptide *K*_*m*_^app^ values for wild-type PRDM7 were determined at a concentration of 1000 μm AdoMet to maintain sufficient assay signal. The AdoMet *K*_*m*_^app^ value for PRDM7 S357Y was determined using a fixed concentration of 50 μm H3K4me2 peptide. AdoMet *K*_*m*_^app^ values for all other mutant proteins were determined using a fixed concentration of 10 μm H3K4me2 peptide. Peptide *K*_*m*_^app^ values for mutant PRDMs were determined using the following AdoMet concentrations: S289N, 1000 μm; S312W, 250 μm; triple mutant, 250 μm; PRDM9 Y357S, 50 μm. Kinetic data from the mutant enzymes were analyzed using a Michaelis-Menten model in GraphPad Prism version 6.05.

##### PRDM7 Overexpression in HEK293 Cells

To examine the methylation of H3K4 and H3K36 in HEK293T cells (ATCC) by PRDM7, we performed an exogenous histone H3/PRDM7 overexpression assay as previously described (33). Briefly, 2 × 10^5^ cells were seeded in 6-well plates in DMEM with 10% FBS and co-transfected with 0.9 μg of FLAG-tagged human *PRDM7* (GenScript, OHu31053) and 0.1 μg of GFP-tagged *histone H3* using 293fectin (Invitrogen) following the manufacturer's instructions. 24 h after transfection, cells were collected and lysed in 50 mm Tris-HCl (pH 8.0), 150 mm NaCl, 2 mm EDTA, 1% Triton X-100, benzonase and protease inhibitors (cOmplete EDTA-free mixture tablets, Roche Applies Science) for 15 min on ice. 1% SDS was added after incubation and lysates were cleared by centrifugation for 15 min at 16,000 × *g*. Protein concentration was determined using BCA Protein Assay Kit (Pierce) and 30 μg of lysate was used for Western blotting via SDS-PAGE, transferred to PVDF membrane, and immunoblotted using the same antibodies for the histone peptide immunoblots as described above, and an anti-FLAG antibody to verify expression of PRDM7 (FLAG-M2, Sigma). Total histone H3 was assessed by blotting using anti-GFP (Clontech, Living Colors® Clone JL-8), which was used as a loading control for the quantifications. Membranes were developed using LI-COR IRDye infrared imaging. Western blots were quantified using ImageJ, with each sample corrected to its GFP loading control, and then normalized to FLAG-empty vector. Experiments were performed 4 independent times and are plotted as the average fold-change relative to control mean ± S.E.

##### Structural Model of PRDM7

The PRDM7 sequence was tethered on the structure of mPRDM9 (mouse PRDM9) in complex with the cofactor product *S*-adenosyl-l-homocysteine and a H3K4me2 peptide (PDB code 4C1Q), and the energy of the system was minimized in the internal coordinates space with ICM (34).

##### Pairwise Sequence Alignments

Sequence alignments of the PRDM7 and PRDM9 constructs were performed using the EMBOSS Needle Online Pairwise Sequence Alignment Tool. The SET domain of PRDM7 and PRDM9 were defined per the Uniprot annotation (residues 244–358 for both proteins). The alignment was performed using a BLOSUM62 matrix using a gap open penalty of 10, a gap extension penalty of 0.5, with no end gap penalty.

## Results

### 

#### 

##### PRDM7 Is an Active Histone Methyltransferase

**FIGURE 2.**
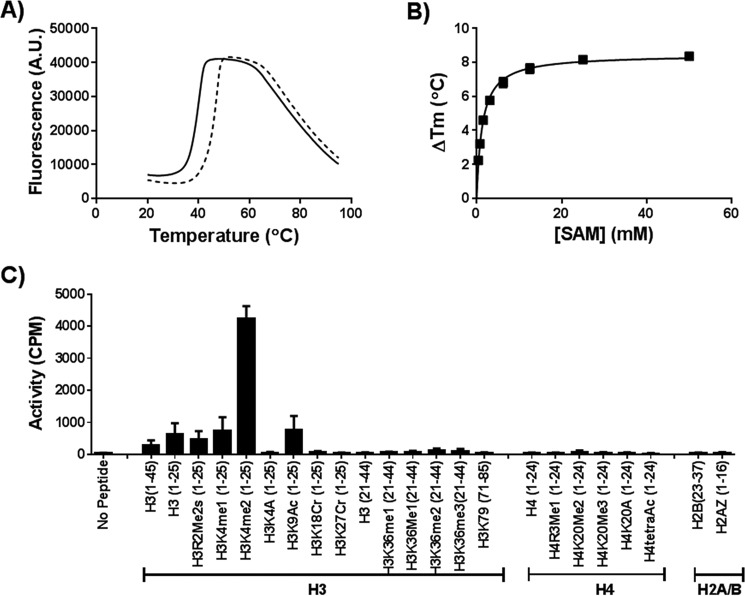
**Wild-type PRDM7 is an active methyltransferase.** *A,* PRDM7 is thermally stabilized by AdoMet (*dashed line*) relative to vehicle control (*solid line*), as shown by differential scanning fluorimetry. *B,* AdoMet (*SAM*) stabilizes PRDM7 in a concentration-dependent manner. *C,* PRDM7 is a trimethylase that is selective for H3K4me2. Peptide screening was performed with 1 μm wild-type PRDM7 and 5 μm test peptide as described under “Experimental Procedures.” Data in *panel A* are presented as representative raw traces. Data in *panels B* and *C* are presented as the mean ± S.D. from at least three independent experiments.

**FIGURE 3.**
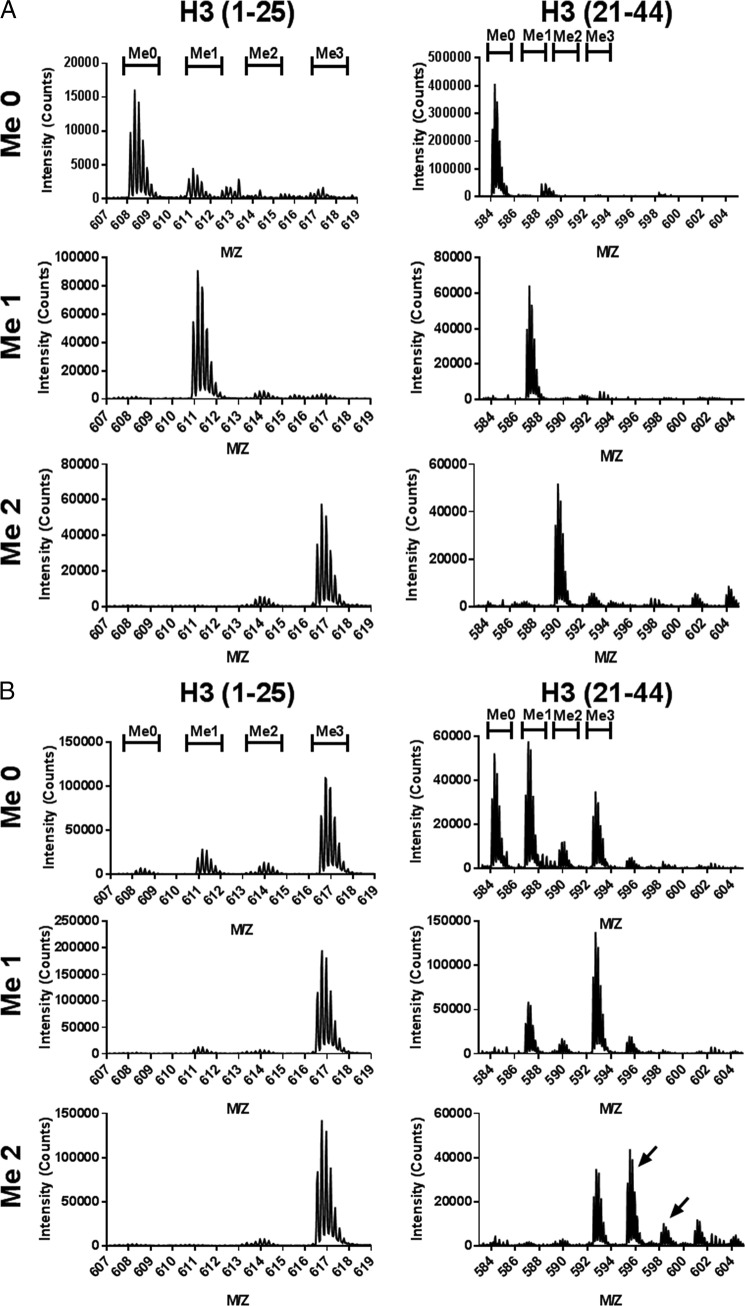
**PRDM7 selectively trimethylates H3K4me2.** Methylation of H3(1–25) peptides or H3(21–44) peptides by (*A*) wild-type PRDM7 (1 μm) or (*B*) S357Y PRDM7 (0.1 μm) as determined by LC-MS-TOF. Spectra shown are the *m*/*z* signal for the +5-charged peptides. Initial K4 methylation states of the substrate peptide are marked on the *left side* and the expected *m*/*z* range for the various product methylation states are indicated at the *top*. For H3K36me2 methylation by PRDM7 S357Y additional peaks corresponding to the addition of 2 or 3 methyl groups was also observed (indicated by *arrows*). Data are presented as a representative single trace from a series of at least three independent experiments.

**FIGURE 4.**
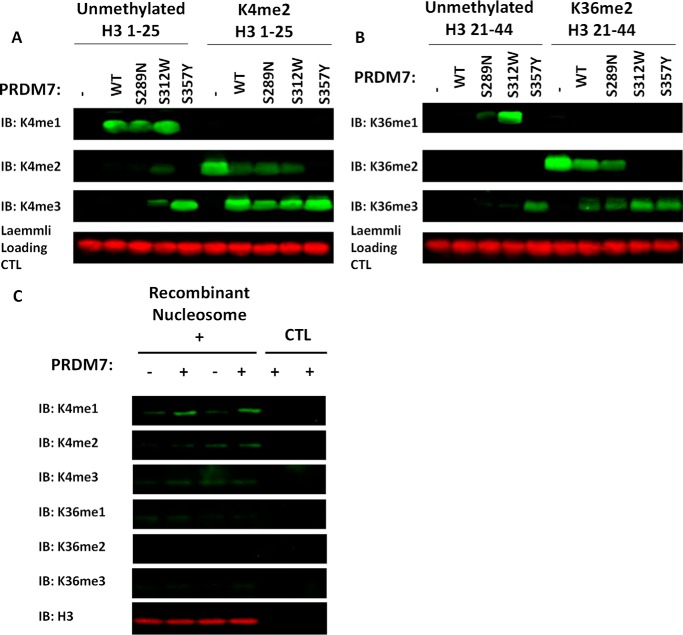
**Western blotting analysis of PRDM7 methylation products.** Analysis of PRDM7 methylation products by Western blotting analysis using (*A*) H3(1–25) peptides and (*B*) H3(21–44) peptides, and (*C*) recombinant nucleosome. Data are presented as a representative blot from at least three independent experiments. *IB*, immunoblot.

**FIGURE 5.**
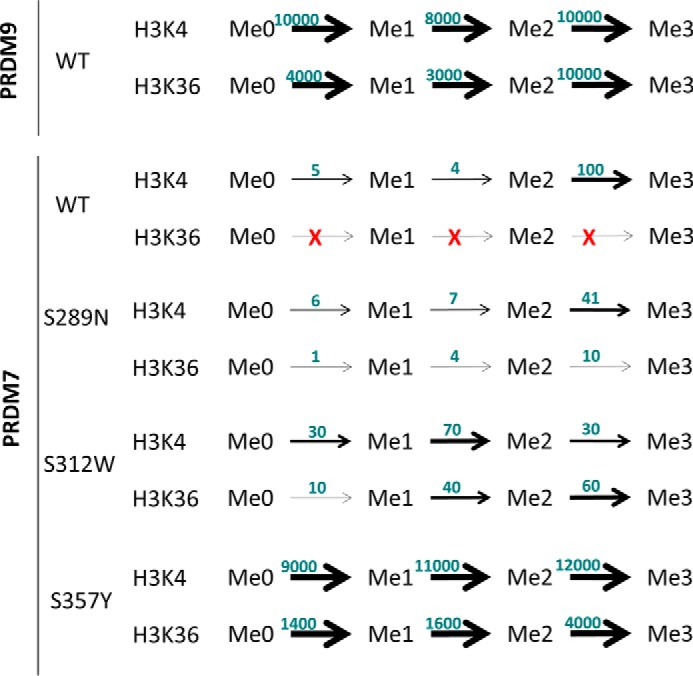
**Graphical overview of the functional consequences of mutation of the divergent residues in the PR domain between PRDM7 and PRDM9.** All *k*_cat_^app^ values in Table 1 have been normalized to trimethylation of H3K4me2 by wild-type PRDM7 as 100, and represented in this figure. PRDM9 data are adapted from the work of Eram and colleagues (14).

**FIGURE 6.**
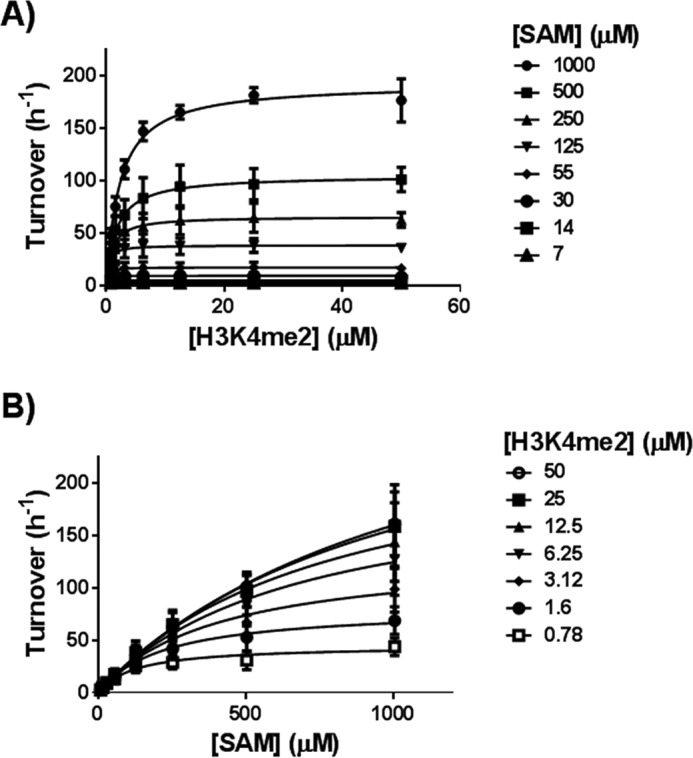
**Determining the kinetic parameters for PRDM7.** Data used to approximate PRDM7 wild-type kinetic parameters for (*A*) H3K4me2 and (*B*) AdoMet (*SAM*) using a bi-substrate matrix assay format. Data were analyzed using the enzyme kinetics module of SigmaPlot (version 11.0). Data are presented as the mean ± S.D. from three independent experiments.

The amino acid sequences of PRDM7 and PRDM9 are 97% identical across the PR domains (residues 244 to 358; Fig. 1). Therefore, PRDM7 was expected to show methyltransferase activity, perhaps with a substrate specificity and level of activity similar to PRDM9. We set out to fully characterize its enzymatic activity *in vitro* and in cells. We first tested its ability to bind to AdoMet using differential scanning fluorimetry (Fig. 2, *A* and *B*). AdoMet stabilized PRDM7 in a concentration-dependent manner, suggesting that PRDM7 retained the ability to bind AdoMet. Subsequently, we screened a library of 23 histone peptides including peptides with different methylation states of H3K4 and H3K36 using a radiometric assay to identify potential PRDM7 substrates. Although active with H3K4me0 and H3K4me1 as substrates, PRDM7 showed significantly higher activity with H3K4me2, indicating it is a more efficient H3K4 trimethylase (Fig. 2*C*). It was also inactive with the H3K4A(1–25) peptide, confirming that Lys-4 is the only methylation site up to residue 25 on the histone H3 tail. As expected, no activity was observed with histone H4, H2A, or H2B peptides. Surprisingly, no activity was observed with any of the peptides harboring H3K36. These findings were further investigated by mass spectral analysis (Fig. 3*A*) and Western blotting analysis (Fig. 4) of reaction products confirming H3K4me2 as the best substrate for PRDM7 with no significant activity on H3K36. Assay conditions were optimized with regards to common additives and pH (data not shown). To ensure accuracy of the kinetic parameters, the linearity of initial velocities for kinetic studies were also confirmed (data not shown). Full kinetic characterization of PRDM7 activity confirmed that it is much more active in trimethylating H3K4me2 (*k*_cat_ of 190 h^−1^) than monomethylation of H3K4me0 (*k*_cat_ of 9 ± 0.9 h^−1^) or dimethylation of H3K4me1 (*k*_cat_ of 8 ± 0.5 h^−1^) with overall 5× higher catalytic efficiencies (Table 1, and Figs. 5 and 6). PRDM7 showed no activity in radiometric assays with H3K36me0, H3K36me1, or H3K36me2 with up to 250 nm enzyme, 900 μm AdoMet, and 50 μm peptide for a period of 1 h.

**TABLE 1:** **Apparent kinetic parameters for PRDM7 and PRDM9 mutants**

Substrate	PRDM7 WT		PRDM7 S357Y		PRDM7 S289N		PRDM7 S312W		PRDM7 Triple Mutant		PRDM9 Y357S	
	*K*_*m*_^app^	*k*_cat_^app^	*K*_*m*_^app^	*k*_cat_^app^	*K*_*m*_^app^	*k*_cat_^app^	*K*_*m*_^app^	*k*_cat_^app^	*K*_*m*_^app^	*k*_cat_^app^	*K*_*m*_^app^*^a^*	*k*_cat_^app^*^a^*
	μ*m*	*h*^−*1*^	μ*m*	*h*^−*1*^	μ*m*	*h*^−*1*^	μ*m*	*h*^−*1*^	μ*m*	*h*^−*1*^	μ*m*	*h*^−*1*^
**AdoMet**	900*^a^*	190*^a^*	14 ± 4	20,100 ± 4,100	200 ± 30	70 ± 4	56 ± 2	67 ± 6	25 ± 2	11,000 ± 2,000	8	0.8
**H3K4me0(1–25)**	0.8 ± 0.1	9 ± 0.9	0.17 ± 0.02	17,300 ± 1,000	0.4 ± 0.2	11 ± 2	0.4 ± 0.1	50 ± 17	1.7 ± 0.6	21,000 ± 5,000	0.1	0.6
**H3K4me1(1–25)**	0.7 ± 0.05	8 ± 0.5	0.3 ± 0.07	21,600 ± 2,100	0.2 ± 0.02	13 ± 1	1 ± 0.1	120 ± 14	2 ± 0.2	27,000 ± 1,000	0.4	0.7
**H3K4me2(1–25)**	3.5 ± 0.4	190*^a^*	0.26 ± 0.06	23,200 ± 4,500	4 ± 0.7	75 ± 10	2 ± 0.3	60 ± 15	3.3 ± 0.5	15,000 ± 2,100	0.1	0.8
**H3K36me0(21–44)**	NA*^b^*	NA	0.4 ± 0.1	2,500 ± 300	1 ± 0.5	2 ± 0.4	0.7 ± 0.4	25 ± 10	1.3 ± 0.4	13,000 ± 3,500	0.5	0.5
**H3K36me1(21–44)**	NA	NA	0.7 ± 0.1	2,900 ± 400	0.3 ± 0.2	8 ± 1	0.7 ± 0.04	77 ± 7	0.7 ± 0.3	10,500 ± 2,000	0.3	0.4
**H3K36me2(21–44)**	NA	NA	3.0 ± 0.3	6,600 ± 700	1 ± 0.3	19 ± 2	5 ± 1	110 ± 45	7 ± 0.4	24,000 ± 1,000	3	2

*^a^* Approximate value. For H3 21–44, H3K36me1 and H3K36me2 experiments for wild-type PRDM7, no methylation was observed when 250 nm enzyme was incubated with 900 μm AdoMet and 50 μm peptide for a period of 1 h. Data are presented as the mean ± S.D. from at least three independent experiments.

*^b^* NA, not active.

##### The PRDM7 PR Domain Is Catalytically Distinct from PRDM9

**FIGURE 7.**
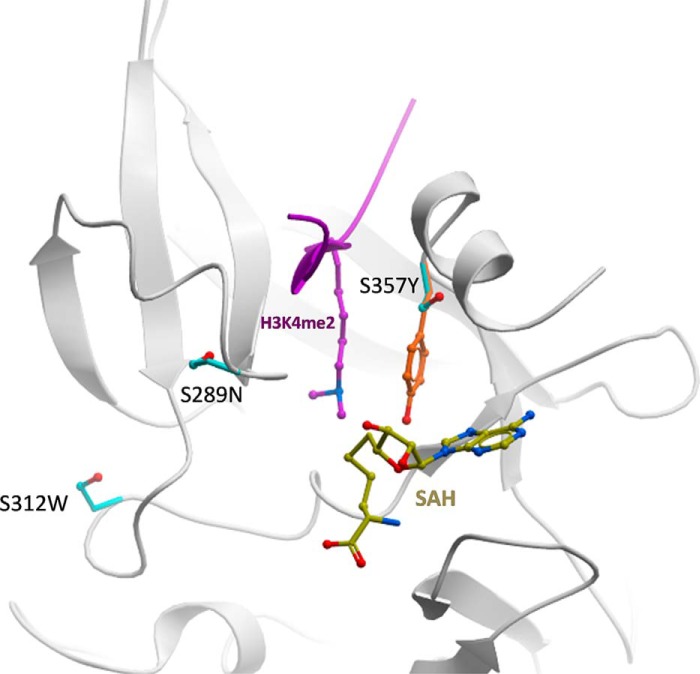
**Mapping of non-conserved side chains on a structural model of PRDM7.** The three PRDM7 serine side chains (*cyan*) that are not conserved in PRMD9 are mapped on a model of PRDM7 (*white ribbon*) built by homology to a mPRDM9 structure in complex with *S*-adenosyl-l-homocysteine (SAH) (*yellow*) and a H3K4me2 peptide (*magenta*). The catalytic tyrosine of PRDM9 (*orange*) is shown.

The PR domain of PRDM7 and PRDM9 only differ by 3 residues (Fig. 1*A*). These residues in PRDM9 correspond to Asn-289, Trp-312, and Tyr-357. In PRDM7 all three have evolved via single point mutations to serine. We hypothesized that the differences in catalytic activity and substrate specificity between PRDM7 and PRDM9 could be a consequence of these single point mutations. Because the crystal structure of PRDM7 has not been determined, we generated a homology model of PRDM7-H3K4me2 peptide based on the PRDM9 structure (PDB code 4C1Q, Fig. 7). This model predicts that: 1) Ser-289 is close to the substrate binding site but does not make direct interactions with the substrate. It could have limited indirect effects on substrate binding, but the fact that a serine is also present at this position in mPRDM9 argues against this possibility. 2) Ser-312 is not at the peptide substrate binding site and does not participate in the sequence-specific recognition of residues flanking the methylated lysine. 3) Ser-357 is occupying the position of the conserved SET domain catalytic tyrosine (structurally aligned with Tyr-357 in mPRDM9 (PDB code 4C1Q) or Tyr-1154 in G9a) (35). Loss of this tyrosine is expected to reduce the nucleophilicity of the departing methyl group of AdoMet, and have a strongly negative impact on catalytic efficiency.

To dissect the role of these serine replacements in PRDM7, we individually mutated each residue back to its corresponding ancestral PRDM9 residue and characterized the biochemical activity of these proteins (Table 1). Wild-type PRDM7 has a very high apparent *K_m_* for AdoMet (900 μm) using H3K4me2 as substrate, and with this assay system we can only approximate the AdoMet *K_m_* value. PRDM7 S289N and S312W have apparent AdoMet *K_m_* values that are intermediate between the wild-type proteins (200 ± 30 and 56 ± 2 μm, respectively). The apparent AdoMet *K_m_* of PRDM7 S357Y (14 ± 4 μm), however, is substantially lower than that for even wild-type PRDM9 (140 ± 9 μm) (14) and this effect is maintained in the PRDM7 triple mutant (S289N/S312W/S357Y; 25 ± 2 μm) (Table 1). Furthermore, the catalytic efficiency (*k*_cat_/*K_m_*) of PRDM7 S357Y (90000 h^−1^ μm^−1^) is dramatically higher than that of wild-type PRDM7 (54 h^−1^ μm^−1^) or S289N (20 h^−1^ μm^−1^) and S312W (30 h^−1^ μm^−1^) mutants, suggesting that this tyrosine to serine mutation dramatically altered the catalytic competency of PRDM7 relative to PRDM9.

The methyltransferase activity and substrate specificity of the PRDM7 mutants was determined by mass spectrometry (Fig. 3*B*), Western blotting (Fig. 4), and radiometric methyltransferase assays (Tables 1 and 2). Using end point mass spectral or Western blotting analysis with saturating concentrations of substrate and AdoMet, wild-type PRDM7 selectively methylated H3K4me2 to H3K4me3 (Fig. 3*A*), but is also capable of mono- and dimethylation at reduced catalytic efficiency (Table 1). Under similar conditions, both PRDM7 S312W and S289N lose the dimethylated substrate specificity and instead gain the ability to methylate H3K36 similar to, but less efficiently than, PRDM9 (Table 1, Fig. 5). Unlike wild-type PRDM7, the S357Y mutant is capable of fully methylating both H3K4 and H3K36 (Table 1, Figs. 3*B* and 5). Western blotting analysis of reaction products confirmed the mass spectrometry data on H3K4 methylation specificity consistent with kinetic data (Fig. 4*A*). Western blotting experiments also showed traces of H3K36 trimethylation by wild-type PRDM7 (Fig. 4*B*). From this, we concluded that wild-type PRDM7 harbors some residual affinity for H3K36 and related peptides used for *in vitro* assays. However, we were not able to measure significant H3K36 methylation activity on peptides for wild-type PRDM7 by radiometric assay, which can be explained by the difference in detection limits of the two assays. The data also indicated that S357Y not only efficiently trimethylated H3K36me2 but is also a better H3K4 trimethylase than wild-type PRDM7 (Table 1, Fig. 4*A*). Mass spectra for S357Y mutant activity using H3K36me2(21–44) as substrate, although not quantitative, shows extra peaks in addition to that for trimethylation, indicating that other residues on this peptide may have been methylated (Fig. 3*B*). However, this may have been a consequence of using a high concentration (100 nm) of enzyme in sample preparation for mass spectrometry than that for enzyme assay (0.5 nm) and may not be physiologically relevant. In radiometric assays using lower concentrations of protein (5 nm), PRDM7 S357Y is selective for H3K4 and H3K36 and has a substrate profile similar to wild-type PRDM9 (Table 1). S357Y PRDM7 had no activity with H3K36me3(21–44) and H3K4A(1–25) peptides (data not shown). Using recombinant nucleosome as substrate only a very low level of activity was observed for native PRDM7, which was detectable only by Western blot (Fig. 4*c*) but not by radiometric assay.

**TABLE 2:** **Swapping the tails of PRDM7 and PRDM9** Apparent kinetic parameters were determined for two additional PRDM7 and PRDM9 mutants, where the tail of PRDM9 (residues 368–385) was swapped with corresponding residues of PRDM7 (7 Tail; residues 368–392 of PRDM7) and vice versa. Data are presented as the mean ± S.D. from at least three independent experiments.

Substrate	PRDM7 (9 tail)		PRDM9 (7 tail)	
	*K*_*m*_^app^	*k*_cat_^app^	*K*_*m*_^app^	*k*_cat_^app^
	μ*m*	*h*^−*1*^	μ*m*	*h*^−*1*^
**AdoMet**	15 ± 1.5	8 ± 0.8	24 ± 1.3	10,800 ± 700
**H3K4me0(1–25)**	0.1 ± 0.01	6 ± 0.2	4 ± 0.5	30,300 ± 2,700
**H3K4me1(1–25)**	0.1 ± 0.02	10 ± 1	5 ± 0.3	26,500 ± 800
**H3K4me2(1–25)**	0.3 ± 0.04	7 ± 0.4	6 ± 1	19,500 ± 2,500
**H3K36me0(21–44)**	0.3 ± 0.1	4 ± 0.4	3 ± 1	17,700 ± 2,200
**H3K36me1(21–44)**	0.1 ± 0.03	6 ± 0.5	0.7 ± 0.06	7,200 ± 40
**H3K36me2(21–44)**	0.7 ± 0.1	14 ± 0.6	15 ± 2	35,000 ± 4,000

Although the PR domains of PRDM7 and PRDM9 are quite similar, the region immediately downstream of their respective PR domains differs substantially (PRDM7(368–392) and PRDM9(368–385); Fig. 1). To determine whether this region plays a role in substrate specificity of the enzymes, we generated constructs that contained the PR domain of PRDM7 or PRDM9 appended with the C-terminal extension of the other protein based upon the constructs used in the assay. Replacing the region of PRDM9 immediately following the PR domain with that of PRDM7 only modestly altered activity relative to wild-type PRDM9 (Table 2). However, appending the C-terminal region of PRDM9 to the PRDM7 PR domain significantly decreased its H3K4 trimethylation activity (*k*_cat_ of 7 ± 0.4 h^−1^), decreased the AdoMet *K_m_* and allowed methylation of H3K4 and H3K36 (Table 2). These data suggest that regions outside of the SET domain may also significantly contribute to the stability or conformation of the active site in PRDM7. Interestingly, the Y357S PRDM9 mutant dramatically lost activity with both H3K4(1–25) and H3K36(21–44) peptides as substrate indicating the active sites of PRDM7 and PRDM9 are significantly different (Table 1).

##### PRDM7 Selectively Increases the Level of H3K4me3 in Cells

**FIGURE 8.**
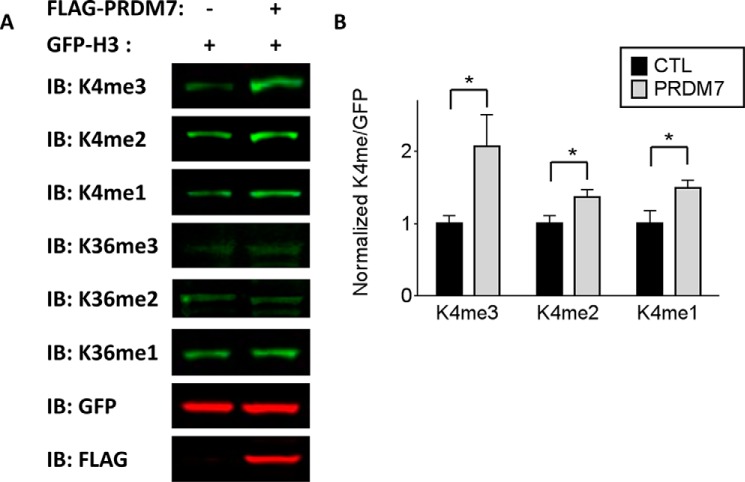
**Overexpression of PRDM7 in cells increases H3K4 methylation of exogenous H3.** *A,* Western blot of HEK293 cells co-transfected with GFP-H3 and FLAG-empty vector or FLAG-PRDM7, respectively. The blots shown are representative for *n* = 4 experiments and quantified in *B* for the H3K4 marks. The quantifications show a stronger increase of H3K4me3 mark. Data represents the methylated/unmethylated ratios (methyl/GFP) normalized to each respective empty vector control. Data shown are *n* = 4 ± S.E., *, *p* < 0.05. *IB*, immunoblot.

Overexpression of full-length human PRDM7 in HEK293 cells increased the level of mono-, di-, and trimethylated H3K4 on exogenously expressed histone H3 (Fig. 8). Although this effect is observed for mono- and dimethyl-H3K4, the increase in methylation is strongest for H3K4 trimethylation. The level of H3K36 methylation remains unaltered in these cells, consistent with the biochemical evidence that PRDM7 is a selective H3K4 trimethyltransferase.

## Discussion

There is little known about the biological role of PRDM7 other than that it is a primate-specific paralog of PRDM9 (30). Like many gene duplications, it is reasonable to surmise that PRDM7 has evolved a novel function independent of its predecessor. This hypothesis is supported by the observation of higher genomic conservation surrounding the *PRDM9* locus than the *PRDM7* locus, suggesting that the *PRDM7* locus has more biological ”freedom“ in which to develop novel functionalities (30). Furthermore, the genomic and protein domain organization of PRDM9 more closely resembles that of the PRDM7/9 co-orthologs from other vertebrates (30), further supporting a conserved activity of PRDM9. These data suggest that PRDM7 may have acquired a novel, primate-specific biological function that is currently unknown. In an attempt to better understand the biochemical properties of PRDM7, we have characterized the differences in methyltransferase activities between the PR domains of PRDM7 and PRDM9 using the known substrate for PRDM9, histone H3.

There are three mutations in the PR domain of PRDM7 that differentiate it from its ancestral paralog. These acquired mutations have allowed PRDM7 to evolve into a distinct HMT with altered substrate specificity. PRDM7 specifically performs di- to trimethylation of H3K4, although only weakly methylating unmodified or monomethylated H3K4. *In vitro*, the catalytic activity of PRDM7 is substantially less than that of PRDM9, an effect that is at least in part due to a very high apparent *K_m_* for AdoMet. However, when compared with other histone methyltransferases, PRDM7 is still a relatively robust methyltransferase *in vitro*, with *k*_cat_ values on the order of 200 h^−1^. Two of the divergent residues between the PR domains of PRDM7 and PRDM9, N289S and W312S (from PRDM9 to PRDM7, respectively), are located on the periphery of the PR domain and appear to possess structural roles that dictate substrate selectivity and modestly affect catalytic efficiency. The third divergent residue, Y357S, has the greatest effect on catalytic function. This residue is a conserved catalytic tyrosine required for formation of a carbon-oxygen hydrogen bond required for AdoMet activation of SET domain methyltransferases (36) and as a structural component of the lysine substrate binding pocket. In PRDM9 this tyrosine residue is important for catalysis by making direct contact with both substrate and cofactor. Corresponding tyrosine residues are commonly present in other SET domain containing methyltransferases (36). In the methyltransferase SETD7, the side chain hydroxyl of this tyrosine makes a carbon-oxygen hydrogen bond with AdoMet to activate the labile methyl group, whereas the hydrophobic portion of the side chain forms van der Waals interactions with the alkyl portion of the substrate lysine (36). Analysis of the mPRDM9 crystal structure in complex with *S*-adenosyl-l-homocysteine and a histone peptide reveals a similar arrangement of tyrosine 357, consistent with this residue playing a similar role in the methylation reaction (PDB code 4C1Q (37)). The corresponding Ser-357 of PRDM7 is too far from AdoMet to activate its departing methyl group, and would require a substantial alteration in the active site to compensate for the lack of the bulky tyrosyl side chain. Additionally, this residue is critically positioned at the entrance of the post-SET domain, a structurally dynamic secondary element that shapes the substrate binding groove (35). Proper positioning of the catalytic tyrosine of SET domain PMTs is believed to contribute in the stabilization of the substrate binding site (38). Mutation of tyrosine to a serine could indirectly affect substrate recognition. This mutation accounts for the majority of the differences in AdoMet apparent *K_m_* and substrate selectivity we observe between PRDM7 and PRDM9. Notably, the *in vitro* AdoMet apparent *K_m_* of wild-type PRDM7 is quite high, substantially higher than the 20–80 μm intracellular AdoMet concentration (39). Despite this, exogenously expressed full-length human PRDM7 is capable of methylating H3K4 of exogenously expressed histone H3 in cells. These data suggest that the cellular concentrations of AdoMet are sufficient to support methylation by the PRDM7 PR domain in the context of a full-length protein in an overexpression system.

Although this study has focused on the PR domain of PRDM7, there are other differences between PRDM7 and its ancestral forbearer that bear mentioning. PRDM9 is only expressed in germ cells where it is essential for meiotic recombination (15), whereas PRDM7 is more broadly expressed in non-meiotic tissue (30). Mice lacking PRDM9 are sterile due to gametogenic failure (15, 22) and two studies have identified single nucleotide polymorphisms in *PRDM9* that may be linked to azoospermia in humans (8, 40). Indeed, *PRDM9* was identified as the first hybrid sterility gene in mammals (25). This and subsequent work has brought forth the hypothesis (41) that PRDM9 may be a major speciation factor (22, 42). The sterility effects of PRDM9 ablation derive from the inability to properly localize the double-stranded DNA breaks required during meiotic recombination (15), a function that requires the zinc finger motifs of PRDM9 and that are fine-tuned by the substantial allelic variation observed in this region of the protein (43). The major isoform of human PRDM9 contains 14 zinc finger moieties, whereas PRDM7 contains either 0 or 4, depending on the isoform (30). Although the biological role of PRDM7 has yet to be determined, the loss in number and complexity of the zinc finger domains suggests that they might not be as important for function of PRDM7 as they are for PRDM9. Furthermore, the divergent region immediately downstream of the PRDM7/PDRM9 PR domains also appears to play a role in the enzymatic activity of the enzymes. Given the differential expression patterns of PRDM7 *versus* PRDM9 and their divergent enzymatic activity, it is unlikely that PRDM7 is biologically redundant with PRDM9.

We report here the first biochemical characterization of PRDM7, the product of a recent gene duplication of PRDM9. PRDM7 selectively performs the trimethylation of histone H3 lysine 4 and has little enzymatic activity upon the other known PRDM9 substrate, H3 lysine 36. In general, the catalytic efficiency of PRDM7 is reduced relative to PRDM9, likely due to the replacement of a conserved tyrosine in the active site of PRDM9 with a serine in PRDM7. Although little is known about the biological function of PRDM7, it appears that it is an active histone methyltransferase with a distinct substrate specificity from PRDM9. As it is only present in primates, genetic ablation of PRDM7 has not been studied. However, it is reasonable to surmise that the phenotype would be substantially different from a PRDM9 knock-out based upon the differences in catalytic efficiency and tissue localization between the two proteins (30). Future work will be focused upon determining the biological role of this unique member of the PRDM family.

## Author Contributions

L. B. designed and performed *in vitro* experiments, analyzed data, and wrote the manuscript. E. L. F. designed and performed Western blotting and in cell experiments, analyzed data, and contributed to writing the manuscript. E. G. purified the proteins. M. S. E. contributed to enzyme assays. P. L. cloned the constructs. C. H. A. reviewed data and contributed to experimental design. M. S. generated the homology model, analyzed data, and contributed to writing the manuscript. M. V. designed experiments, analyzed and reviewed data, supervised the project, and wrote the manuscript.

## Footnotes

3 The abbreviations used are:

PRDM: PRDI-BF1 and RIZ homology domain
Bis-tris propane: 1,3-bis[tris(hydroxymethyl)methylamino]propane
AdoMet: *S*-adenosylmethionine.
